# Genomics Research: World Survey of Public Funding

**DOI:** 10.1186/1471-2164-9-472

**Published:** 2008-10-10

**Authors:** Jennifer Reineke Pohlhaus, Robert M Cook-Deegan

**Affiliations:** 1 Centre for Genome Ethics, Law & Policy, Institute for Genome Sciences & Policy, Duke University, Durham, North Carolina, USA

## Abstract

**Background:**

Over the past two decades, genomics has evolved as a scientific research discipline. Genomics research was fueled initially by government and nonprofit funding sources, later augmented by private research and development (R&D) funding. Citizens and taxpayers of many countries have funded much of the research, and have expectations about access to the resulting information and knowledge. While access to knowledge gained from all publicly funded research is desired, access is especially important for fields that have broad social impact and stimulate public dialogue. Genomics is one such field, where public concerns are raised for reasons such as health care and insurance implications, as well as personal and ancestral identification. Thus, genomics has grown rapidly as a field, and attracts considerable interest.

**Results:**

One way to study the growth of a field of research is to examine its funding. This study focuses on public funding of genomics research, identifying and collecting data from major government and nonprofit organizations around the world, and updating previous estimates of world genomics research funding, including information about geographical origins. We initially identified 89 publicly funded organizations; we requested information about each organization's funding of genomics research. Of these organizations, 48 responded and 34 reported genomics research expenditures (of those that responded but did not supply information, some did not fund such research, others could not quantify it). The figures reported here include all the largest funders and we estimate that we have accounted for most of the genomics research funding from government and nonprofit sources.

**Conclusion:**

Aggregate spending on genomics research from 34 funding sources averaged around $2.9 billion in 2003 – 2006. The United States spent more than any other country on genomics research, corresponding to 35% of the overall worldwide public funding (compared to 49% US share of public health research funding for all purposes). When adjusted to genomics funding intensity, however, the United States dropped below Ireland, the United Kingdom, and Canada, as measured both by genomics research expenditure per capita and per Gross Domestic Product.

## Background

Genomics research, as a field of study, is largely a creature of the past two decades and is generally defined as the study of whole genomes. The term genomics came into common use in 1987 to distinguish "high throughput" data- and technology-intensive approaches to studying DNA structure and function from the more established approach of studying DNA structure and function of individual genes. The history of genomics research is embedded in the Human Genome Project and its parallel private sector components. In 2003, with the completion of a high-quality sequence of the human genome, the National Human Genome Research Institute (NHGRI) of the National Institutes of Health (NIH) announced that "the genomic era is now a reality" [1]. Genomics has become central to biomedical and disease-based research; genomic technologies are being used to identify genetic factors involved in nine of the ten leading causes of death in the United States (excluding accidents; [2,3]).

As the social and personal implications of genomics research have become apparent (its power to identify an individual uniquely, to influence the health care decisions of some, and to inform the study of genealogy and ancestry of individuals and populations), the public has become increasingly interested in understanding genomics. Genomics is being used in many populations for purposes such as large genome-wide population studies, personalized genomics, and genomic ancestry tests. Genomics has become an issue of interest to the general public, and an element of current culture, as evidenced by several articles in the popular press [4-11]. As genomics is incorporated into health care [12], law enforcement [13], ancestry tracing [14], and other activities, feelings of hope and fear surrounding individual genomic sequencing have emerged [15] and public funding allocated to genomics research has increased [evidenced by the establishment of the NIH Center for Human Genome Research, which later became the National Human Genome Research Institute; [16]].

Most countries provide public funding for scientific research, under some variation of a mission to promote or improve the nation's health. Many countries are investing in genomics as an element of biotechnology, and as a pathway to economic development. The rise of genomics funding results from the priority that governments have placed on such research, which is influenced by policy decisions. One of the foremost issues in genome research policy is allocation of funds and research prioritization [17]. Research prioritization is determined by stakeholders with varying perspectives; therefore each country, and in fact each organization, is likely to arrive at a different set of priorities, and subsequent allocation of resources.

The individuals who determine research priorities and subsequent allocation of funds for each organization are accountable to the donors who provided funding support; in the public sector, most research funding is derived from taxpayer support. Research funding in the United States is distributed by Congress, whose Members are accountable to citizens and constituents. As "genomics is beginning to bring understanding that everyone is at risk for something based on their genes" [18], public interest in genomics research increases. Both those advocating for health research funding to address diseases or health conditions, and policy-makers who determine government resource allocations can use information about funding levels to inform advocacy positions and governmental funding decisions.

To facilitate its input, the public needs access to data, benchmarks, and indicators of current research funding, past funding trends, future projections, and comparisons with funding organizations around the world. Several organizations study and publish such data and benchmarks for general R&D funding or health funding, including the R&D Budget and Policy Program of the American Association for the Advancement of Science (AAAS) [19], the Division of Science Resources Statistics at the National Science Foundation (NSF) [20] and its National Science Board's Science and Engineering Indicators [21], the Statistics Portal of the Organization for Economic Cooperation and Development (OECD) [22] and the Global Forum on Health Research [23]. While the authors of "Monitoring Financial Flows in Health Research" characterize their estimates as "very rough," they remain the best statistics available on global heath research [24]. Occasional studies have included reporting on genomics research, including the 2004 "Financial Flows" report [see Highlight 2.1 (page 27) in [25]], which cited a previous survey (in 2000) that our current survey builds upon. There has been no update specific to genomics since 2000 [26], however, so we undertook this "world survey" to update and expand that survey. We report the allocation of public funds that countries and organizations in the public sector (governments, nonprofit organizations, and international organizations) provide for genomics research. This survey of public sector funding provides patterns and trends of worldwide genomics funding, allowing for initial comparisons across organizations and countries, and complements an effort to estimate genomics expenditures by private companies [27].

In our survey, we simply asked each organization to provide a quantitative estimate of the amount of genomics research they funded each year; we did not supply a definition of genomics research (because a universally agreed-upon definition does not exist). To facilitate participation of as many organizations as possible, we encouraged organizations to use their own definitions of genomics, so that funding data could be easily extracted from budget information. Some funding organizations did not have a standard definition of genomics research or requested a specific definition. In those cases, we replied that genomics research is defined by research on the entire genome of an organism instead of research on individual genes and gene functions [See Discussion and Chandrasekharan et. al [27] for a more complete definition and taxonomy]. Organizations that were unable to provide estimates for genomics research were not included in the study. This survey does not cover all the organizations or all the countries that publicly fund genomics research. As for any funding table, there is definitional wobble and incomplete reporting. For reasons elaborated below, however, we believe most of the major sources of public funding have been identified and that the funding figures provided here can inform researchers and policymakers on the state of genomics research, at least as rough indicators and for information about trends.

## Results

For this world survey of genomics research, we identified 89 different organizations, representing 26 countries and 7 international organizations (covering multi-country regions; see Additional File). An initial response was received from about half (42; or 48 if counting those that we did not contact but about which we received information), with most of those (34) supplying the results shown in Table 1. The 14 organizations that responded to our request but did not provide information cited reasons such as the information not being available in the format requested (i.e. they were unable to estimate funding allocated to genomics research) or that genomics was not a part of their research portfolio.

**Table 1:** Genomics Funding by Organization and Year, in US$ (millions)

**Rank**	**Organization**	**2003**	**2004**	**2005**	**2006**	**Source**
n/a	Department of Health and Human Services (DHHS) National Institutes of Health (NIH) – GENETICS^O^	$4236	$4535	$4840	$4878	[28]

1	NIH: National Cancer Institute (NCI) + National Human Genome Research Institute (NHGRI)	$562	$593	$593	$571	See Tables 2, 3 and text

2	European Commission^J^	$459	$462	$466	$468	Personal Communication, Indridi Benediktsson, September 2006

3	European Commission Matching Funds^J^	$459	$462	$466	$468	Personal Communication, Indridi Benediktsson, September 2006

4	United Kingdom Wellcome Trust^O^	$194	$194	$208	$199	[52,53]

5	Department of Energy (DOE) Office of Biological and Environmental Research^O^	$129	$152	$154	$158	[33-36]

6	National Science Foundation (NSF) Biological Sciences Directorate^O^	$124	$129	$134	$141	Personal Communication, Vernon Ross, April 2007

7	Japan Ministry of Education, Culture, Sports, Science and Technology (MEXT)^A^	$84.5	$99.2	$119	$125	Personal Communication, Kazuko Shinohara, January 2007

8	United Kingdom Biotechnology and Biological Sciences Research Council (BBSRC)^A^	$121	$127	$128	$117	Personal Communication, Clare Nixon, January 2007

9	Genome Canada^A^	$67.3	$65.7	$71.6	$106	Personal Communication, Genny Cardin, July 2006

10	China (Ministry of Science and Technology, National Natural Science Foundation of China, and Chinese Academy of Sciences)^J^	$80	$80	$80	no report	Personal Communication, Anonymous, October 2007

11	Germany Nationales Genomforschungsnetz (NGFN)^J^	$71.6	$61.4	$62.0	$64.8	Personal Communication, Uta Strasser, September 2006

12	Department of Defense (DOD) Congressional Directed Medical Research Programs (CDMRP)^O^	$102	$86.8	$53.5	$54.9	[45]

13	Cancer Research UK^A^	$34.1	$45.2	$48.1	$51.0	Personal Communication, Lynne Davies, January 2007

14	Netherlands Genomics Initiative^J^	$48.8	$18.1	$51.7	$45.8	[31,87,88]

15	South Korea Ministry of Science and Technology (MOST)^J^	$39.7	$35.0	$41.5	$44.3	Personal Communication, Jeongheui Lim, January 2007

16	United States Department of Agriculture (USDA) Agricultural Research Service^O^	$32.5	$38.8	$41.9	$43.1	Personal Communication, Peggy DelCollo and Joe Garbarino, November 2006

17	Ireland Higher Education Authority^J^	$34.1	$34.7	$34.5	$34.5	Personal Communication, Sorcha Carthy, August 2006

18	Canada Natural Sciences and Engineering Research Council (NSERC)^A^	$28.2	$30.4	$32.2	$34.1	Personal Communication, Barney Laciak, October 2007

19	Department of Homeland Security (DHS)^O^	$13.4	$25.9	$32.8	$27.2	Personal Communication, Elizabeth George, November 2006

20	Japan Ministry of Agriculture, Forestry and Fisheries (MAFF)^A^	$17.9	$22.9	$21.7	$21.7	Personal Communication, Kazuko Shinohara, January 2007

21	Canadian Biotechnology Strategy (CBS)^A^	$16.1	$15.9	$15.9	$16.2	[55]

22	Howard Hughes Medical Institute (HHMI)^J^	$15.2	$13.8	$14.3	$15.6	Personal Communication, Sherry White, August 2006

23	Canada National Research Council (NRC) Genomics and Health Initiative^A^	$18.2	$15.8	$13.4	$15.3	Personal Communication, Gary Fudge, August 2007

24	Japan Ministry of Health, Labour and Welfare (MHLW)^A^	$12.8	$13.8	$18.6	$14.9	Personal Communication, Kazuko Shinohara, January 2007

25	American Cancer Society^J^	$5.95	$6.90	$5.61	$11.9	Personal Communication, Donella Wilson, July 2006

26	Spain Genoma Espana^J^	$9.98	$12.1	$13.5	$11.7	Personal Communications, Javier Montero Plata, June and September 2006

27	Australia National Health and Medical Research Council (NHMRC) *	$7.54	$6.34	$6.24	$7.65	Personal Communication, Marian Blake, July 2006

28	Department of Health and Human Services (DHHS) Centers for Disease Control and Prevention (CDC)^O^	$3.85	$4.53	$6.99	$6.95	[37,38]

29	Department of Defense (DOD) Defense Advanced Research Projects Agency (DARPA) Bio/Info/Micro Program^O^	$34.0	$13.4	$13.2	$6.5	[41-44]

30	South African Medical Research Council^J^	$1.73	$1.84	$2.04	$2.24	Personal Communication, Clive Glass, October 2007

31	Ireland Science Foundation^J^	$7.41	$7.96	$1.38	$2.24	Personal Communication, Tracy Moloney, September 2006

32	Ireland Health Research Board^J^	$1.29	$2.23	$1.50	$1.61	Personal Communication, Gillian Hastings, January 2007

33	South Africa National Research Foundation^J^	$0.658	$0.560	$0.999	no report	Personal Communication, Marna van Rooyen, October 2006

34	Japan Ministry of Economy, Trade and Industry (METI)^A^	$0	$4.51	$0	$0	Personal Communication, Kazuko Shinohara, January 2007

	**TOTAL**	**$2834**	**$2878**	**$2948**	**$2881**	

Genomics research funded, by organization, is shown in millions of US$ per year. The total funding values each year were determined by summing the values from each organization, with the exception of the first row (NIH – Genetics), which is described in the text. Rankings were determined by ordering the 2006 values, where the average of the three previous years was used as a substitute for 2006 values when the actual 2006 data was unavailable. The start of each fiscal year is indicated by the superscript character after each organization, where J = January 1, A = April 1, and O = October 1.

*Although the fiscal year for the Australian government begins July 1, the original data was reported by calendar year.

An estimate for worldwide genomics funding from the responding organizations averages around US$2.9 billion for 2003 – 2006 (Table 1). Although the table is incomplete for 2006 due to unavailability of funding amounts for South Africa's National Research Foundation and China, their combined total averaged less than three percent of worldwide government and nonprofit genomics research in 2003 – 2005.

When beginning this survey, we noted that the United States NIH publicly reported an estimate of funding for the research area of genetics [28]. To find out more about this reporting practice, we requested and received the breakdown of genetics research funding by NIH component (Table 2; Personal Communication, Arlette Howard, May 2006). In an effort to determine whether the NIH considered genomics research to be a subset of genetics research, we requested the definition of genetics research from the Office of Budget and found that the NIH does "not have an official definition" (Personal Communication, Arlette Howard, May 2006). Turning to each of the 24 grant-issuing Institutes and Centers of the NIH, we requested information about their definitions for genetics and genomics research. Except for the National Cancer Institute (NCI) and the NHGRI, discussed below, each Institute and Center informed us that they did not have a definition for genetics, and that they do not track genomics research funding.

**Table 2:** Genetics Funding by the National Institutes of Health, in US$ (thousands)

	**Fiscal Year 2003**	**Fiscal Year 2004**	**Fiscal Year 2005**	**Fiscal Year 2006, Est**	**Fiscal Year 2007, Bdgt**
National Cancer Institute	$1,275,213	$1,364,851	$1,451,384	$1,451,384^a^	$1,435,419^a^

National Human Genome Research Institute	$453,105	$475,735	$468,049	$463,900	$459,039

National Institute of General Medical Sciences	$430,175	$455,079	$441,168	$438,079	$433,698

National Institute of Neurological Disorders and Stroke	$354,940	$400,140	$416,474	$414,113	$409,972

National Institute of Child Health and Human Development	$308,304	$320,310	$329,252	$329,600	$329,400

National Institute of Mental Health	$194,484	$201,564	$239,907	$237,849	$235,625

National Heart, Lung and Blood Institute	$253,240	$229,478	$230,335	$231,487	$232,644

National Aging Institute	$47,305	$52,273	$201,852	$200,300	$198,300

National Institute of Environmental Health Sciences	$169,231	$201,603	$199,125	$197,532	$195,754

National Eye Institute	$176,028	$182,542	$198,605	$197,413	$195,242

National Center for Research Resources	$157,993	$163,381	$165,859	$167,551	$166,890

National Institute of Dental and Craniofacial Research	$123,596	$136,914	$138,279	$136,752	$134,469

National Institute on Alcoholism and Alcohol Abuse	$105,805	$108,196	$111,369	$110,255	$109,263

National Institute of Arthritis and Musculoskeletal Disorders	$74,813	$78,071	$79,568	$78,775	$77,985

National Institute on Deafness and Other Communications Disorders	$51,474	$52,253	$57,266	$56,715	$56,170

National Institute on Drug Abuse	$39,142	$48,858	$52,036	$51,724	$51,465

Roadmap		$38,712	$39,421	$39,421	$39,421

National Institute of Biomedical Imaging and Bioengineering	$9,157	$9,932	$8,975	$8,885	$8,823

Office of the Director	$2,338	$7,085	$5,165	$4,754	$4,764

National Institute of Nursing Research	$5,642	$4,600	$3,537	$3,509	$3,477

Fogarty International Center	$3,712	$2,905	$1,978	$1,978	$1,978

National Center on Minority Health and Health Disparities	$62	$289	$260	$259	$257

National Center on Complementary and Alternative Medicine					

National Institute of Allergy and Infectious Diseases					

National Institute of Diabetes and Digestive and Kidney Disorders					

National Library of Medicine					

Total NIH Genetics	$4,235,759	$4,534,771	$4,839,864	$4,822,235^b^	$4,780,055^b^

The amount of genetics research funded by NIH components (24 grant-issuing Institutes and Centers, the Office of the Director, and the Roadmap) is indicated in thousands of US$; Fiscal year 2006 is an estimate, and fiscal year 2007 is the President's budget. The values in this table were provided by the NIH Office of Budget (Personal Communication, Arlette Howard, May 2006).

^a ^NCI actual genetics funding for fiscal years 2006 and 2007 was $1,530,200 and $1,523,628, respectively, in thousands (Personal Communication, Weston Ricks, July 2008).

^b ^Total NIH Genetics funding (actual) for fiscal years 2006 and 2007 was $4,878,000 each year, in thousands [28].

The NCI provided a definition for both genetics and genomics, and informed us that fiscal year 2006 marked the first year of data collection on genomics research (Personal Communication, Weston Ricks, June 2006). In fiscal years 2006 and 2007, NCI genomics research funding corresponded to 7.0% and 10.2% of genetics funding, respectively (Personal Communication, Weston Ricks, July 2008), as indicated in Table 3. To estimate the amount of genomics research funded by NCI in fiscal years 2003 – 2005, we applied the average fraction from 2006 – 2007 (8.6%) to the amount of genetics funding (Table 3). Upon examining the funding history of NHGRI [29], we observed that the NHGRI genetics values from the NIH Office of Budget (Table 2) corresponded to the amount of the NHGRI total budget, less Roadmap Transfer, and Research Management & Support. Since this value also corresponds to Intramural Research plus all (Extramural) Research, Training, and R&D Contracts for Human Genome Project [29], and since its mission "encompasses a broad range of studies aimed at understanding the structure and function of the human genome and its role in health and disease" [30], this amount appears to be the figure that NHGRI reports as genomics research, reflected in Table 3. Although other Institutes and Centers support genomics research, they do not report the values, so we present the values from NCI and NHGRI as a minimum estimate for NIH spending on genomics research (US$563 – 571 million in 2003 – 2006).

**Table 3:** Genomics Funding by the National Institutes of Health, in US$ (thousands)

	**FY 2003**	**FY 2004**	**FY 2005**	**FY 2006**	**FY 2007**
National Cancer Institute (NCI)^c^	$109,598	$117,302	$124,739	$107,413	$154,944

National Human Genome Research Institute (NHGRI)^d^	$453,105	$475,735	$468,049	$463,550	$468,232

NCI+NHGRI	$562,703	$593,037	$592,788	$570,963	$623,176

The amount of genomics research funded by the National Cancer Institute (NCI) and the National Human Genome Research Institute (NHGRI), in thousands of US$, where FY = fiscal year.

^c ^NCI values for fiscal years 2006 and 2007 were 7.02% and 10.17% of genetics funding, respectively (Personal Communication, Weston Ricks, July 2008). We estimated the values for fiscal years 2003 – 2005 by multiplying the genetics funding values for each year by the average ratio of genomics:genetics funding for fiscal years 2006 – 2007.

^d ^We determined NHGRI values as the total NHGRI funding appropriation minus Roadmap Transfer and Research Management & Support [[29]; see text].

The 34 organizations in the survey account for 13 countries directly, as well as indirectly including another 28 countries that are eligible for full funding by the European Commission (27 member states of the European Union plus Iceland, Norway, Liechtenstein, Switzerland, Israel, and Turkey; Personal Communication, Indridi Benedikttson, September 2006). Only five of the thirty-three countries fully eligible for European Commission funding are directly represented in this world survey (the United Kingdom, the Netherlands, Germany, Ireland, and Spain). Of note, funds from the European Commission cover about 50% of the costs of the funded projects; the remaining costs are covered by each institution that receives funding (Personal Communication, Indridi Benedikttson, September 2006), which are included in Table 1 as European Commission matching funds.

We grouped the organizations by country (Table 4) into three tiers based on the amount of genomics research funded. The five countries or regions that funded more than US$100 million in genomics research in each of the four years surveyed are the United States, Other Europe, the United Kingdom, Canada, and Japan. The next tier includes China, Germany, the Netherlands, South Korea, and Ireland, which reported between US$35 – 80 million in genomics research funding each year (with the exception of 2004 for the Netherlands, which was lower than the other years because expenditures were "declared (much) later" [31]). Finally, Spain, Australia, and South Africa reported less than US$14 million each year.

**Table 4:** Genomics funding by country or region, in US$

**Rank**	**Country**	**2003**	**2004**	**2005**	**2006**
1	United States	$1,023,269,552	$1,064,204,394	$1,049,343,544	$1,035,309,134

n/a	Other Europe	$918,484,501	$923,787,529	$931,315,483	$936,768,150

2	United Kingdom	$349,339,913	$366,753,082	$384,375,015	$366,638,731

3	Canada	$124,919,355	$122,960,000	$128,400,000	$166,260,163

4	Japan	$115,289,855	$140,488,722	$159,359,375	$161,451,613

5	China	$80,000,000	$80,000,000	$80,000,000	$0*

6	Germany	$71,602,210	$61,409,396	$61,947,905	$64,800,000

7	Netherlands	$48,775,731	$18,127,090	$51,743,764	$45,793,541

8	South Korea	$39,713,000	$35,000,000	$41,495,000	$44,341,000

9	Ireland	$42,841,306	$44,848,081	$37,366,395	$38,332,844

10	Spain	$9,981,553	$12,079,692	$13,544,247	$11,668,897

11	Australia	$7,547,002	$6,386,677	$6,243,976	$7,655,910

12	South Africa	$2,388,907	$2,397,169	$3,037,712	$2,238,932*

The amount of genomics research funded by each country or region is indicated in US$. The region "Other Europe" refers to funding in European countries for which the table does not directly account (i.e. excludes the funding in other rows of the table that was supplied directly from public funds in the listed European countries), and thus is not included in the rank. Rankings were determined by ordering the 2006 values, where the average of the three previous years was used as a substitute for 2006 values when the actual 2006 data was unavailable. Data is reported by fiscal year, as indicated in Table 1.

* The 2006 data is incomplete because of missing information from the South African National Research Foundation or from China's research organizations.

To examine the priority each country places on genomics research, as a measure of "genomics intensity," we normalized public funding for genomics research to the national population (Table 5) and to Gross Domestic Product (GDP; Table 6). Ireland spent approximately US$9 – 11 on genomics research per capita while the United Kingdom, Canada, the United States, and the Netherlands funded between US$2.8 – 6.4 per capita each year (with the exception of 2004 for the Netherlands, see above). Japan, South Korea, and Germany spent between US$0.70 – 1.30 annually per capita on genomics, while the remaining four countries surveyed (Australia, Spain, South Africa, and China) spent less than US$0.38 annually per capita. Genomics funding as a fraction of GDP shows the same relative ranking as genomics funding per capita for the first five countries, followed by South Korea, Japan, China, Germany, South Africa, Australia, and Spain (Table 6).

**Table 5:** Genomics funding per capita by country, in US$

**Rank**	**Country**	**2003**	**2004**	**2005**	**2006**
1	Ireland	$10.77	$11.09	$9.05	$9.13

2	United Kingdom	$5.87	$6.13	$6.41	$6.06

3	Canada	$3.94	$3.85	$3.98	$5.10

4	United States	$3.52	$3.62	$3.54	$3.46

5	Netherlands	$3.01	$1.11	$3.17	$2.79

6	Japan	$0.90	$1.10	$1.25	$1.26

7	South Korea	$0.83	$0.73	$0.96	$0.91

8	Germany	$0.87	$0.74	$0.75	$0.79

9	Australia	$0.38	$0.32	$0.31	$0.37

10	Spain	$0.24	$0.28	$0.31	$0.26

11	South Africa	$0.05	$0.05	$0.06	$0.05*

12	China	$0.06	$0.06	$0.06	$0.00*

The amount of genomics research funded per capita by each country or region is indicated in US$. Rankings were determined by ordering the 2006 values, where the average of the three previous years was used as a substitute for 2006 values when the actual 2006 data was unavailable. Data is reported by fiscal year, as indicated in Table 1.

* The 2006 data is incomplete because of missing information from the South African National Research Foundation or from China's research organizations.

**Table 6:** Genomics funding per GDP by country (×100,000)

**Rank**	**Country**	**2003**	**2004**	**2005**	**2006**
1	Ireland	27.27	24.44	18.61	17.48

2	United Kingdom	19.25	17.02	17.24	15.55

3	Canada	14.38	12.37	11.34	13.06

4	United States	9.34	9.09	8.42	7.81

5	Netherlands	9.04	2.98	8.21	6.91

6	South Korea	6.53	5.15	5.27	5.05

7	Japan	2.72	3.06	3.49	3.62

8	China	4.88	4.14	3.58	0.00*

9	Germany	2.93	2.24	2.22	2.24

10	South Africa	1.44	1.12	1.27	0.87*

11	Australia	1.43	1.00	0.88	1.03

12	Spain	1.13	1.16	1.20	0.96

The amount of genomics research funded per GDP by each country or region is indicated in US$ (× 100,000 for ease of comparison). Rankings were determined by ordering the 2006 values, where the average of the three previous years was used as a substitute for 2006 values when the actual 2006 data was unavailable. Data is reported by fiscal year, as indicated in Table 1.

* The 2006 data is incomplete because of missing information from the South African National Research Foundation or from China's research organizations.

## Discussion

This survey does not cover all the organizations or countries that publicly fund genomics research. To estimate how well the values reported in this survey correspond to the actual public funding of genomics research in each country, we determined, where possible, the research funders for each country that were not included in this survey (see Additional File 1).

### Analysis by Country/Region

#### United States

**Figure 1:**
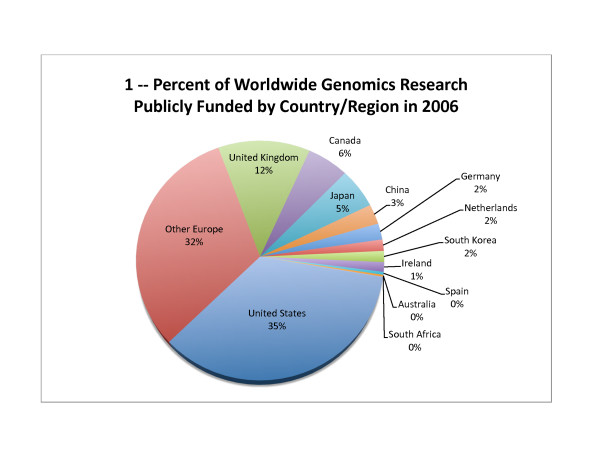
**Percent of Worldwide Genomics Research Publicly Funded by Country/Region in 2006**. The percent of worldwide genomics funding provided by each identified country or region is depicted graphically for 2006, except for South Africa and China, where the average of the three previous years was used as a substitute for 2006 values, since 2006 data was unavailable.

**Figure 2:**
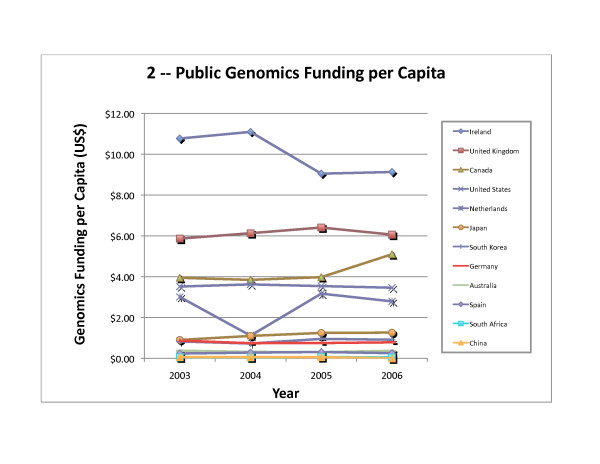
**Public Genomics Funding per Capita**. Genomics funding per capita for 2003 – 2006, as shown in Table 5, is depicted graphically.

**Figure 3:**
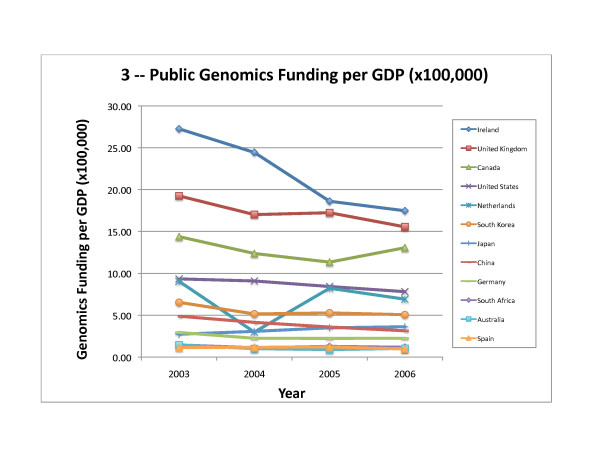
**Public Genomics Funding per GDP (×100,000)**. Genomics Funding per GDP for 2003 – 2006, as shown in Table 6, is depicted graphically.

The United States funded about one third of the genomics research reported in this world survey for 2003 – 2006 (Figure 1 for 2006; compared to 49% of worldwide government and nonprofit funding for health research in 2001 [25]). The amount of funding and funding per capita rose slightly in 2004 but decreased in 2005 and 2006 (Table 4, Table 5 and Figure 2), while funding per GDP steadily decreased (Table 6 and Figure 3).

Over half of the United States contribution was provided by the NIH. As described above, we made considerable effort to determine the amount of genomics research funding by each Institute and Center, but we were only able to quantitate NCI and NHGRI directly. In these two cases, the amount of genomics research compared to genetics research was just under 10% (NCI) and 100% (NHGRI). Actual kinds of research undoubtedly differ between NCI and NHGRI, but a significant portion of the difference is likely attributable to different definitions and reporting practices for "genomics" as a category. If other Institutes and Centers fund genomics in proportion to genetics research similar to the NCI proportion, the additional genomics expenditure (for the Institutes and Centers that reported Genetics funding) would total $215 – 250 million in Table 2.

Although the NIH funded US$4.2 – 4.9 billion of genetics research (about 16 – 17% of the total NIH budget, and the sixth or seventh most funded research area of 210 categories, [28]) we note missing data from four grant-issuing Institutes and Centers (National Center on Complementary and Alternative Medicine, National Institute of Allergy and Infectious Diseases, National Institute of Diabetes and Digestive and Kidney Disorders, and National Library of Medicine; Table 2). A simple query of CRISP (Computer Retrieval of Information on Scientific Projects), a searchable database of biomedical research projects funded by the NIH and other Department of Health and Human Services (DHHS) components, reveals that each of these NIH components does fund genetics and genomics research [32]. Thus, the actual amount of *genetics *research is likely to be higher than that reported by NIH, and the additional amount of *genomics *research could be much more than $215 – 250 million.

The Department of Energy (DOE) Office of Biological and Environmental Research funded genomics research in the Life Sciences funding category under the subcategories of Microbial Genomics, Genomics: GTL [Genomes To Life program], Human Genome, and Functional Genomics/Health Effects [33-36]. The NSF Biological Sciences Directorate funded genomics research under the following three programs: Microbial Genome Sequencing Program, the Plant Genome Project, and the 2010 Project (Personal Communication, Vernon Ross, April 2007).

Genomics funding from the Centers for Disease Control and Prevention (CDC) increased from US$3.85 million (2003) to US$6.95 million (2006) [37,38]; part of this increase was due to a change in budget structure in 2005, when genomics funding moved from environmental health to health promotion, with slightly different conventions in calculating funding values (Personal Communication, Sara Schmit, July 2006). The United States Department of Agriculture (USDA) funded genomics research projects through the Agricultural Research Services (Personal Communication, Peggy Del Collo and Joe Garbarino, November 2006). The DHS supported genomics research through the Science and Technology Directorate (Personal Communication, Elizabeth George, November 2006). The Howard Hughes Medical Institute (HHMI) funded genomics research through its Science Department at US$13 – 15 million (Personal Communication, Sherry White, August 2006). The American Cancer Society provided between US$6 – 12 million in genomics research funding (Personal Communication, Donella Wilson, July 2006).

The US Department of Defense supports research and development through the Defense Advanced Research Projects Agency (DARPA), which pursues research and technology where risk and payoff are both very high [39], and through the United States Army with the Congressionally Directed Medical Research Program (CDMRP) [40]. DARPA sponsored genomics research under the Bio/Info/Micro Sciences programs: Biocomputational Systems and Bio Interface, and the Comparative Genomics for National Security Goals [41-44]. We used the online database of the Congressionally Directed Medical Research Program to determine the funding for genomics research by searching for awards that were classified in the "genomics and proteomics" research topic (2003 and 2004) or the "genomics" research topic (2005 and 2006) or awards that listed "genom%" in the abstract, where% is a wild character [45].

To estimate the completeness of the United States spending on genomics research reported here, we determined the percentage of federal and nonprofit funding on R&D that is covered by the entities in this survey. Of the US$1,023 – 1,064 million per year in federally funded research and development in all disciplines (2003 – 2006), six of the entities included in this survey (DOD, DOE, NIH, NSF, USDA, and DHS) accounted for 84 – 86% [46]. Although we were unable to determine the percentage of total research and development funds provided by the nonprofits listed in our survey, the overall contribution of nonprofits towards total research and development funding in the US was 3% in 2004 [47]. Combined, these two data indicate that there are unlikely to be major public funders in the United States that are not addressed in this survey, and that the survey accounts for a large majority of total genomics government and nonprofit expenditures in the United States. The remaining public funds for genomics research are likely to be attributable to the government and nonprofit funders identified in this survey that did not supply data, such as the four NIH Institutes and Centers named above, the National Aeronautics and Space Administration (NASA) Ames Center, National Institutes of Standards and Technology (NIST), National Oceanic and Atmostpheric Administration (NOAA), the J. Craig Venter Institute and the Institute for Genome Research (TIGR), and the Bill & Melinda Gates Foundation.

#### European Union

The European Commission funds genomics research in the European Union, plus Iceland, Norway, Liechtenstein, Switzerland, Israel, and Turkey. In the Sixth Framework Programme of the European Commission, covering 2002 – 2006, the major funding areas that included genomics research were Advanced Genomics and its Applications for Health, and Combating Major Diseases [Personal Communication, Indridi Benediktsson, September 2006; [48]]. European Commission funds, including matching funds, provided about 32% of worldwide public genomics research funding in 2006 (Figure 1). Recently, a new European agency was created (the European Research Council) that will be an "independent, quality-driven funding body run by the scientists themselves" [49] and will likely increase the public funding of genomics (along with other types of scientific) research in Europe.

In an effort to include the European countries funded through the European Commission but not otherwise covered in this world survey, we contacted organizations from Austria (Fonds zur Forderung der Wisenschaftlichen Forschung), Denmark (Ministry of Science, Technology and Innovation), Estonia (Estonian Genome Foundation), Finland (Academy of Finland), France (Association Francaise Contre les Myopathies, La Recherche Agronomique au Service des Pays du Sud, Centre National de la Recherche Scientique, GenHomme, l'Institut National de la Recherche Agronomique, Institut Pasteur, and Institut de Recherche pour le Developpement), Hungary (National Office for Research and Technology), Iceland (Rannis), Italy (Agenzia Spaziale Italiana), Poland (Ministry of Scientific Research and Information Technology), and Sweden (Knut and Alice Wallenberg Foundation), but we received no response from any of these except for the Academy of Finland and Centre National de la Recherche Scientique (see Additional File 1), both of which provided an initial response before communication ceased. We also contacted several international organizations active in Europe (European Space Agency, NordForsk, and the Human Frontier Science Program), but were unsuccessful in gathering funding data.

#### United Kingdom

Spending on genomics research in the United Kingdom decreased slightly in 2006, after a steady increase from 2003 to 2005 (Table 4). The United Kingdom was the second highest funder of genomics research per capita and per GDP (Tables 5 and 6); both measures of genomics "intensity" declined in 2006 (Figures 2 and 3). The three United Kingdom public funders in this survey are Cancer Research UK, the Wellcome Trust, and the Biotechnology and Biological Sciences Research Council (BBSRC), each of which allocated at least 10% of its research monies for genomics. An analysis of the annual reports of the BBSRC shows that 35 – 40% of the total research funded (through responsive research grants, core strategic grants, and research initiatives) was genomics [50,51]. Similarly, the Wellcome Trust reported spending 29 – 38% of its grant expenditures on genomics research [52,53]. Finally, the genomics research funded by Cancer Research UK was estimated at 10% of its charitable expenses (Personal Communication, Lynne Davies, January 2007).

#### Canada

Canada supplied about 6% of total genomics research funding in 2006 (Figure 1), which increased significantly from a relatively constant contribution in 2003 – 2005 (Table 4). Likewise, the genomics intensity measures (genomics funding per capita and per GDP) increased in 2006 (Tables 5 and 6). Its public funding on genomics research is likely to continue increasing, since it recently began a new initiative, the Public Population Project in Genomics [54].

Four Canadian organizations are included in this survey: Genome Canada, the Natural Sciences and Engineering Research Council (NSERC) of Canada, the National Research Council (NRC) and the Canadian Biotechnology Strategy (CBS). The CBS includes six departments and agencies that are building capacity for new research (Agriculture and Agri-Food Canada, Department of Fisheries & Oceans, Department of Natural Resources Canada, Environment Canada, Health Canada, and National Research Council (NRC) [55]), although the contribution from NRC (Canadian $6 million per year) was reduced to avoid double-counting of NRC-funded genomics research (Personal Communication, Gary Fudge, October 2007). Other public funders of genomics research include the Canadian Institutes of Health Research (formerly the Medical Research Council), the Canada Foundation for Innovation, and the Social Sciences and Humanities Research Council, none of these responded to the survey.

#### Japan

Japan's spending on genomics research steadily increased between 2003 and 2006, when it reached 5% of worldwide public funding (Table 4 and Figure 1). Although its ranking dropped from fourth (Table 4) to sixth or seventh when adjusted for intensity (Tables 5 and 6), genomics research is increasing by all measures, indicating that may be poised to overtake some countries in the coming years. In fact, the Second Science and Technology Basic Plan (2001 – 2005) was intended to close the gap between Japan and the other G7 nations in the percentage of R&D funding provided by government. The Third Science and Technology Basic Plan, which began in 2006, has a goal to increase overall government funding of R&D until the Science and Technology investment equals 1% of Japan's GDP [56].

The values reported in this world survey for Japan are likely a near-complete representation of the government's investment in genomics, since the four Japanese ministries reported in this survey (Ministry of Economy, Trade and Industry [METI], Ministry of Education, Culture, Sports, Science and Technology [MEXT], Ministry of Agriculture, Forestry and Fisheries [MAFF], and the Ministry of Health, Labour and Welfare [MHLW]) are the main government funders of life science and genomics research [57-60]. One nonprofit organization, Kazusa DNA Research Institute, was identified and contacted, but did not respond to the survey.

#### China

Our source for China indicated that the central government funded about US$40 million per year of genomics research through the National Natural Science Foundation of China (NSFC), the Chinese Academy of Sciences (CAS), and two programs at the Ministry of Science and Technology (MOST): the National High-tech Development R&D Program (863 Program) and the National Key Basic Research Program (973 Program). The source also indicated that an equal amount of funding was provided by local governments (Personal Communication, Anonymous, October 2007), yielding a total of US$80 million per year – or about 3% of worldwide public genomics research funding (Figure 1). Since our source, a senior official at a publicly funded organization who was not authorized to speak on such issues, preferred to remain anonymous, we made numerous attempts to contact multiple individuals at the above organizations and the United States NSF-Beijing for attributable data; unfortunately, we were unsuccessful in gathering the data via those means.

#### Germany

In Germany, the Federal Ministry for Education and Research (Budesministerium fuer Bildung und Forschung, BMBF) coordinates the R&D initiative for the federal government [61]. Analysis of genomics research funding by Germany, which provided about 2% of the total (Figure 1), revealed that genomics research peaked in 2003; although 2006 levels were slightly higher than 2004 and 2005 levels, by all measures (Tables 4, 5, and 6). The National Genome Research Network (Nationalen Genomforschungsrutz, NGFN), established in 2001 and included in this world survey, is one of two organizations to which BMBF supplied funds for both scientific research and research into the ethical, legal, and economic impacts of genomics [62]. The other organization is the German Human Genome Project [62], which was discontinued in June 2004 [63] and not included in this survey. Another public funder, the German Research Foundation (Deutsche Forschungsgemeinschaft, DFG), which promotes research at universities and other publicly financed research institutions [64], did not respond to the survey.

#### The Netherlands

The primary funder of genomics research in the Netherlands is the Netherlands Genomics Initiative (NGI), which was established by the Netherlands Organization for Scientific Research (Nederlandse Organisatie voor Wetenschappelijk Onderzoek; NWO) and five government ministries in 2001. The NGI was created to combine existing strengths into one initiative composed of core activities (Centres of Excellence, Innovative Clusters, Technology Centres, Exceptional Talent, International Research Consortiums, and Innovate Genomics Clusters) and research programs [31]. Netherlands genomics research funding was about 2% of the total in 2006 (Figure 1); but its rank jumped from seventh (Table 4) to fifth when adjusting for genomics intensity (Tables 5 and 6).

#### South Korea

In South Korea, which accounted for 2% of total genomics research funding in 2006 (Figure 1), the Ministry of Science and Technology (MOST) is the central agency for national science and technology development, including support for basic and applied research and development supported by the government [65], making it likely that the values reported here represent nearly all of the country's genomic research funding. Although South Korea's funding for genomics research has been increasing steadily since 2004, the incoming president has pledged to eliminate four government ministries, possibly merging the MOST with the Ministry of Education, which may weaken it [66].

#### Ireland

According to the Office of Science and Technology of the Ireland Department of Enterprise, Trade, and Employment, three government agencies are responsible for most of the genomics funding in Ireland (Personal Communication, Helen Dixon, August 2006). These three agencies – the Higher Education Authority, the Science Foundation, and the Health Research Board – are included in this survey. The Irish government has recently increased its investment in R&D with the goal of meeting or exceeding the average R&D investment of European Union countries [67]. Indeed, Ireland appears to place a high priority on genomics research since its rank increases dramatically from ninth (Table 4) to first in funding per capita and per GDP (Tables 5 and 6). However, Ireland's spending on genomics research is likely to be overestimated, since the values reported by the Higher Education Authority include all biomedical/bioscience projects (because each program lists some degree of genomics research; Personal Communication, Sorcha Carthy, August 2006). Of note, the Science Foundation indicated that genomics research represents approximately 19% of its bio-related funding to date (Personal Communication, Tracey Moloney, September 2006). Applying the same percentage to the bioscience funding of Higher Education Authority would decrease total genomics research funding in Ireland to US$9.4 – 16.8 million per year, corresponding to US$2 – 4 per capita.

#### Spain

Genoma España is the Foundation for the Development of Genomic and Proteomic Research, and is backed by the Spanish State through the Ministry of Health and Consumer Affairs (Ministerio de Sanidad y Consumo), and the Ministry of Education and Science (Ministerio de Educacion y Ciencia) [68]. Genoma España is one of four main funding centers in biotechnology, along with the Center for Development of Industrial Technology (Centro para el Desarollo Tecnologio Industrial) in the Ministry of Industry, Trade and Tourism (Ministerio de Industria, Turismo y Comercio), the Instituto de Salud Carlos III in the Ministry of Health and Consumer Affairs, and the Ministry of Education and Science [69]. Although Spain is a low-ranked by all measures (Tables 4, 5, and 6), it has recently developed new government programs to increase total R&D from 1.25% of GDP to 2% of GDP by 2010 [69-71], indicating that Spain's overall investment in genomics research is likely to increase.

#### Australia

In Australia, the National Health and Medical Research Council (NHMRC) is the "peak body" for supporting health and medical research [72] and provided US$6 – 8 million for genomics research (2003 – 2006; Table 1). Two other organizations that fund genomics research, the Australian Research Council, and the Australian Genome Research Facility, did not respond to the survey. Australia has initiated a National Collaborative Research Infrastructure Strategy, which will include funding for genomics research [73], and could serve to increase the amount of funding supplied for genomics research, as it currently accounts for less than 1% of the total funding (Figure 1).

#### South Africa

The government agency responsible for supporting basic and applied research in South Africa is the National Research Foundation [74], while most government funding for health research is funded through the Medical Research Council [75]. The amount of genomics research funded by South Africa was US$2.2 – 3.0 million for both of these government funders (2003 – 2006; Table 4); the Centre for Research on Science & Technology did not respond to our survey.

#### New Zealand

The three main funders of genomics research in New Zealand are the Foundation for Research in Science and Technology (FRST), the Royal Society, and the Health Research Council (Personal Communication, Kate McDavitt, August 2006). Only FRST, which represents half of the New Zealand government-funded R&D [76], responded. Unfortunately, although FRST was very willing to provide funding information, staff were unable to segregate their funded research into a category that represented only genomics research, and we could not include them in the tabulation.

#### Middle-income countries with developing genomics sectors

As described by the World Health Organization, genomics research is beginning to occur in developing countries and regions such as Brazil, China, India, and the Asia-Pacific Region [77]. We contacted organizations in Argentina (Agencia Nacional de Promocion Cientifica y Tecnologica, and Consejo Nacional de Investigationes Cientificas y Tecnicas), India (Ministry of Science and Technology Division of Science and Technology), and Russia (Englehardt Institute of Molecular Biology Russian Genome Project), as well as the Asian Technology Information Program (ATIP) (see Additional File) about their funding of genomics research, but we received no response other than an initial response from the ATIP before communication ceased. The difficulty we encountered in acquiring an estimate of genomics research expenditures in China is described above. It will take further work and detailed case studies to develop a complete report of genomics research expenditures that are occurring in middle-income countries and developing regions. One such case study is in progress for Brazil, where public funders include the National Council for Scientific and Technological Development (Conselho Nacional de Desenvolvimento Cientifico e Tecnológico), and the State of São Paolo Research Foundation (Fundação de Amparo à Pesquisa do Estado de São Paulo; FAPESP); preliminary data indicates that FAPESP funded about US$56 million (unadjusted for inflation) of genomics research from 1997 – 2007 [78].

### Private Funding of Genomics Research

This study represents only government and non-profit "public" funders of genomics research. The previous survey in 2000 included private companies, and showed a roughly 2:1 ratio of private genomics R&D to public funding (an estimated $1.658 billion from "public" sources compared to $2.016 billion for publicly traded genomics firms and another $800 million to $1 billion from members of the Pharmaceutical Research and Manufacturers Association [research-intensive pharmaceutical and large biotechnology companies; [26]]). In 2004, an estimated US$3.2 billion was invested in genomics R&D by 270 genomics firms from 25 countries [27]. Similar to the public funding of genomics research described here, top countries for private genomics firms are the United States, Canada, Germany, France, United Kingdom, and Japan [27].

### Caveats

There are two major caveats to consider when interpreting the figures reported in this world survey. First, many organizations and countries are not included in this study, either because we did not know to contact them or because they did not respond. Thus, the full amount of genomics research funded by public sources is certainly higher than the estimates reported in this study.

Second, the values provided by each organization are estimates, according to each organization's own definition of genomics research, which is not uniform across all organizations. For example, organizations might have included some genetics research in their genomics research estimates. Consider the NHGRI and the CDC definition of genomics as "the study of all of [a person's genes]" including "interactions of those genes with each other and the person's environment" [2,79]). Interestingly, another US government agency, the Environmental Protection Agency, although not queried in this world survey, provides a definition of *genomics*, "the study of genes and their function" [80], that is almost exactly the definition provided for *genetics *by the NHGRI [79] and in the literature [[17,27] and references therein]. We note, however, that we only included values from organizations that were able to respond to our query for genomics research; organizations stating that they were not able to extract information from their funding databases for genomics research were not included.

## Conclusion

Although this survey is an estimate of funding for genomics research, and necessarily fuzzy and approximate, it represents the only attempt to perform such a survey to our knowledge, and therefore provides patterns and trends as a rough indicator for planning among researchers, science administrators, policymakers, and the general public.

This world survey would not have been possible had we tried to gather funding information for genomics research according to a strict and consistent definition. If governments and private funders believe that genomics as a funding category is permanent and worth retaining as a separate category for analysis, it will need a more uniform definition. This is particularly relevant for the NIH, the world's largest biomedical research funder, which, except for two constituent institutes, (the NHGRI and the NCI) does not track funding of genomics research. And these two Institutes report widely different figures for genomics as a fraction of genetics research, reflecting some real differences but probably also reflecting different definitions of genomics research. Furthermore, the closest research category that the NIH does track, genetics, is not defined centrally.

The NIH is in the process of developing a "portfolio analysis" web-accessible tool that will allow the public to access information about NIH projects by research area, the definitions of which will be "laboriously crafted with input from hundreds of scientists" [81]. The effort that is being expended to create this tool indicates the importance that the NIH places upon appropriately categorizing public expenditures by research area. If genetics, genomics, or both are deemed important categories in which to monitor science trends and for budget planning, then it would be worthwhile to invest in a process to clarify definitions, and the NIH's definition might well become a world standard.

The NCI, a component of the NIH, provided a definition of genomics: "the identification, characterization and quantification of all genes involved in a particular pathway, organelle, cell, tissue, organ or organism that can be studied in concert to provide accurate and comprehensive data about that system" (Personal Communication, Weston Ricks, June 2006). This is a detailed definition that encompasses and expands upon the definition of genomics already in use by the NHGRI and the CDC (see above). If the NIH includes genomics as a research area in its new portfolio analysis tool, the NCI definition could serve as a starting point for the development of a trans-NIH definition of genomics research.

Genomics research has become incorporated into scientific and medical research, and is beginning to be applied in medicine and commerce. Genomics captures the attention of the general public because of its technological power to study the structure and function of DNA, with the consequent potential to reveal intimate details about individuals, populations, and associations between genotype and phenotype.

The amount of funding provided for genomics research is of interest to both scientific and lay communities worldwide. This world survey indicates that overall public funding for genomics research, as a minimum estimate, averaged around US$2.9 billion annually (2003 – 2006). Government and nonprofit funding of genomics research is likely to comprise between one-third and one-half of the total funding (where the remainder is for-profit private funding), based on the worldwide distribution of funding for health research (where government and nonprofit funding for health research account for 45% and 7% of worldwide funding for health research, respectively [82], and based on separate surveys of private genomics R&D in 2000 [26] and [27]).

## Methods

This survey was conducted by email and telephone. Government and nonprofit contacts were assembled by contacting known genome research administrators and scientists, adding contacts from public information and genome websites, and building on the previous world survey of genomics research begun in 2000 [26].

Generally, contacts were emailed the survey question, which asked respondents to identify the total amount of funding that their organizations supplied for genomics research each year (2003 – 2006), with the understanding that estimates were acceptable. In some cases, initial contact was by phone. Follow-up email correspondence often occurred, with the most effort being expended on procuring figures from the largest public funders. In a few cases, such as China, we were able to obtain funding information only from intermediaries familiar with science budgets because of their role in national planning and as performers of genomics research.

Data were requested by fiscal year and are reported in the tables for the calendar year that encompassed most of that fiscal year. Specifically, the calendar year at the beginning of the fiscal year was used when the fiscal year began on April 1, while the calendar year at the end of the fiscal year was used when the fiscal year began on October 1. When funding amounts were supplied in a currency other than US$, they were converted to US$, using the purchasing power parity (PPP) indices provided by the OECD [83], except for South Africa, where implied PPP indices were calculated from International Monetary Fund (IMF) data [84].

To determine per capita genomics funding, the amount of funding per country (in US$) was divided by the estimated population in the middle of the year, as provided by OECD for 2003–2005 [85]. The population data for 2006 were gathered from the Population Reference Bureau 2006 Data Sheet for all countries [86]. To determine genomics funding per GDP, the amount of funding per country (in US$) was divided by the GDP (in US$) for each year, provided by the IMF [84].

Rankings listed in the tables were determined by ordering 2006 values, except in the two instances where the 2006 data were not reported. In those cases, an average of the three previous years determined the ranking order for 2006.

## Abbreviations

AAAS: American Association for the Advancement of Science; ATIP: Asian Technology Information Program; BBSRC: Biotechnology and Biological Sciences Research Council (United Kingdom); BMBF: Budesministerium fuer Bildung und Forschung (Germany); CAS: Chinese Academy of Sciences; CBS: Canadian Biotechnology Strategy; CDC: Centers for Disease Control and Prevention (United States); CDMRP: Congressionally Directed Medical Research Program (United States); DARPA: Defense Advanced Research Projects Agency (United States); DFG: Deutsche Forschungsgemeinschaft (Germany); DHHS: Department of Health & Human Services (United States); DHS: Department of Homeland Security (United States); DNA: Deoxyribonucleic Acid; DOD: Department of Defense (United States); DOE: Department of Energy (United States); FAPESP: Fundação de Amparo à Pesquisa do Estado de São Paulo (Brazil); FRST: Foundation for Research in Science and Technology (New Zealand); FY: Fiscal Year; GDP: Gross Domestic Product; HHMI: Howard Hughes Medical Institute (United States); IMF: International Monetary Fund; MAFF: Ministry of Agriculture: Forestry and Fisheries (Japan); METI: Ministry of Economy, Trade and Industry (Japan); MEXT: Ministry of Education, Culture, Sports, Science and Technology (Japan); MHLW: Ministry of Health, Labour and Welfare (Japan); MOST: Ministry of Science and Technology (Japan); NASA: National Aeronautics and Space Administration (United States); NCI: National Cancer Institute (United States); NGFN: National Genome Research Network (Germany); NGI: Netherlands Genomics Initiative; NHGRI: National Human Genome Research Institute (United States); NHMRC: National Health and Medical Research Council (Australia); NIH: National Institutes of Health (United States); NIST: National Institute of Standards and Technology (United States); NOAA: National Oceanic and Atmospheric Administration (United States); NRC: National Research Council (Canada); NSERC: Natural Sciences and Engineering Research Council (Canada); NSF: National Science Foundation (United States); NSFC: National Natural Science Foundation of China; NOW: Nederlandse Organisatie voor Wetenschappelijk Onderzoek (Netherlands); OECD: Organization for Economic Cooperation and Development; PPP: Purchasing Power Parities; R&D: Research & Development; TIGR: The Institute for Genome Research (United States); UK: United Kingdom; US: United States; USDA: United States Department of Agriculture.

## Authors' contributions

JRP generated the research question, contacted the organizations listed in this survey, generated and analyzed the data, and drafted the manuscript. RMCD participated in study design and coordination, contributed the initial contact list from the 2000 World Survey, and helped to draft the manuscript. Both authors read and approved the final manuscript.

## Supplementary Material

Additional file 1 **Identified organisations** Click here for file
